# Exploring causal relationships between circulating inflammatory proteins and superficial thrombophlebitis: A Mendelian randomization study

**DOI:** 10.1097/MD.0000000000050749

**Published:** 2026-09-25

**Authors:** Qingzhi Liu, Weiyue Chen, Youwen Zhang, Xiangyu Wang, Hong Mao, Boxun Chen, Jiaxiang Xu, Yannan Wang

**Affiliations:** a The First School of Clinical Medicine, Shandong University of Traditional Chinese Medicine, Jinan, Shandong, China; b Department of Peripheral Vascular Diseases, Shandong University of Traditional Chinese Medicine Affiliated Hospital, Jinan, Shandong, China; c Department of Emergency Surgery, Shandong University of Traditional Chinese Medicine Affiliated Hospital, Jinan, Shandong, China.

**Keywords:** inflammatory biomarkers, inflammatory proteins, mendelian randomization, superficial

## Abstract

This study explores the potential causal relationship between circulating inflammatory proteins and superficial thrombophlebitis (STP) using Mendelian randomization (MR). We utilized data on 91 circulating inflammatory proteins from publicly available genome-wide association studies and STP data from the FinnGen consortium’s R11 release. The primary analysis method was inverse variance-weighted analysis, complemented by MR-Egger regression and weighted median methods for validation. Additionally, sensitivity analyses and reverse MR tests were conducted to assess the reliability of the results. In the inverse-variance weighted analysis, we observed nominal positive associations between circulating inflammatory proteins, including neurotrophin-3 (NT-3) (OR = 1.132, 95%CI: 1.024-1.252, *P* = .016), osteoprotegerin (OR = 1.120, 95%CI: 1.013–1.238, *P* = .027), and vascular endothelial growth factor A (OR = 1.068, 95%CI: 1.008–1.132, *P* = .027), and the risk of STP (all raw *P* < .05). Conversely, interleukin-12 subunit beta (OR = 0.917, 95%CI: 0.858-0.980, *P* = .011) and interleukin-8 (OR = 0.905, 95%CI: 0.824–0.993, *P* = .035) showed nominal inverse associations with STP risk. However, none of these associations remained statistically significant after Bonferroni or Benjamini-Hochberg FDR correction for multiple testing. Sensitivity analyses showed no significant pleiotropy or heterogeneity, and the effect directions were consistent across complementary MR methods. This hypothesis-generating MR study identified nominal suggestive associations between 5 circulating inflammatory proteins (neurotrophin-3, osteoprotegerin, vascular endothelial growth factor, interleukin-12 subunit beta, and interleukin-8) and STP risk, though none survived correction for multiple testing. These findings provide candidate inflammatory proteins for future validation and underscore the potential role of inflammation in STP pathogenesis.

**Key messages**

This study explores the potential causal relationship between circulating inflammatory proteins and superficial thrombophlebitis (STP).

Utilizing a Mendelian randomization approach, we analyzed 91 circulating inflammatory proteins and found that NT-3, osteoprotegerin and vascular endothelial growth thrombophlebitis factor A were positively associated with the risk of STP, while interleukin-8 (IL-8) and interleukin-12 subunit beta(IL-12B) exhibited protective effects against STP.

As a hypothesis-generating study, these findings identify candidate inflammatory proteins warranting further validation and provide directions for future research on the inflammatory mechanisms of STP. Additionally, given the close relationship between STP and venous ulcers, these observations may also offer insights into the roles of these inflammatory proteins in venous ulcer pathogenesis.

## 1. Introduction

Superficial Thrombophlebitis (STP) is an inflammatory vascular disease that primarily affects the lower extremity veins, particularly the great saphenous vein (60%–80% of cases) and the small saphenous vein (10%–20% of cases), but it can also occur in other locations such as the upper extremities, head and neck, chest and abdominal wall, breasts, and penis.^[1–4]^ Typical clinical manifestations include painful, cord-like structures along the course of the vein, accompanied by redness, warmth, and tenderness, with possible surrounding erythema (skin redness) and edema (tissue swelling).^[5]^ Even after the inflammatory symptoms have subsided, the persistent presence of organized thrombus and venous return obstruction can still lead to long-term chronic hypoxia and inflammation of the skin in the lower extremities, ultimately potentially developing into a venous ulcer. Although STP is traditionally considered a benign and self-limiting disease, recent epidemiological studies have shown that, if not treated effectively, up to 7.3% to 44% of STP patients may develop deep vein thrombosis 20% to 33% may experience asymptomatic Pulmonary Embolism (PE), and 2% to 13% may even suffer from symptomatic PE. Furthermore, STP is also recognized as a risk factor for recurrence of venous thromboembolism following the first episode of deep vein thrombosis or PE.^[6,7]^

Previous studies have demonstrated a close association between circulating inflammatory proteins and the risk of varicose veins.^[8]^ Recent large-scale whole-genome sequencing and genome-wide association studies (GWAS) on venous thromboembolic diseases have further identified novel pathogenic genetic variants and risk loci, and systematically confirmed that inflammatory response and coagulation pathway dysfunction are the core pathogenic mechanisms of venous thrombotic diseases including STP, providing a solid genetic basis for exploring inflammatory targets of this disease.^[9]^ STP, as one of the significant complications of varicose veins, not only shares this intimate link with circulating inflammatory proteins but also often leads to the development of venous ulcers. The inflammation caused by elevated levels of these proteins exacerbates the condition, potentially contributing to skin damage and ulceration. The 3 recognized major factors contributing to the formation of STP are hypercoagulability, slow blood flow, and venous wall injury. Numerous clinical and experimental studies have confirmed that elevated levels of circulating inflammatory proteins, such as interleukin-6, tumor necrosis factor-*α*, and C-reactive protein, are associated with the risk of developing STP.^[10]^ Furthermore, while these 3 factors promote inflammatory responses, circulating inflammatory proteins can also exacerbate hypercoagulability and venous wall injury. However, due to the lack of comprehensive research, the specific causal relationship between circulating inflammatory proteins and STP remains unclear. Therefore, employing alternative methods to further explore the causal relationship between STP and circulating inflammatory proteins, and establishing the inflammatory mechanism as a promising therapeutic target for STP, will pave the way for prevention and treatment approaches for STP.

Mendelian randomization (MR) is a widely used method in epidemiological studies that employs genetic variants as instrumental variables (IVs) to infer whether a particular risk factor causally influences the occurrence of a disease.^[11]^ The core of this method lies in the random assignment characteristic of genetic variants, which means that, in a natural state, alleles are randomly distributed to offspring during fertilization, thus serving as a natural experiment to explore potential causal relationships.^[12]^ In our study, we delved into the potential causal relationship between circulating inflammatory proteins and STP using the MR approach.

## 2. Materials and methods

### 2.1. Study design

This study employed aggregated data from public databases to perform an MR analysis, aiming to explore the causal link between 91 circulating inflammatory proteins and STP. To guarantee data independence and mitigate errors stemming from participant overlap, the data pertaining to exposure and outcome were sourced from separate databases. Additionally, to uphold the reliability and precision of this research, it was crucial to adhere to the 3 fundamental assumptions of MR analysis: a robust correlation between the IVs and the exposure; mutual independence between the instrumental variables (IVs) and potential confounding factors; and the IVs can solely exert an influence on the outcome via the exposure. We present an overview and the hypotheses of the MR design employed in this study (Fig. 1).

**Figure 1.**
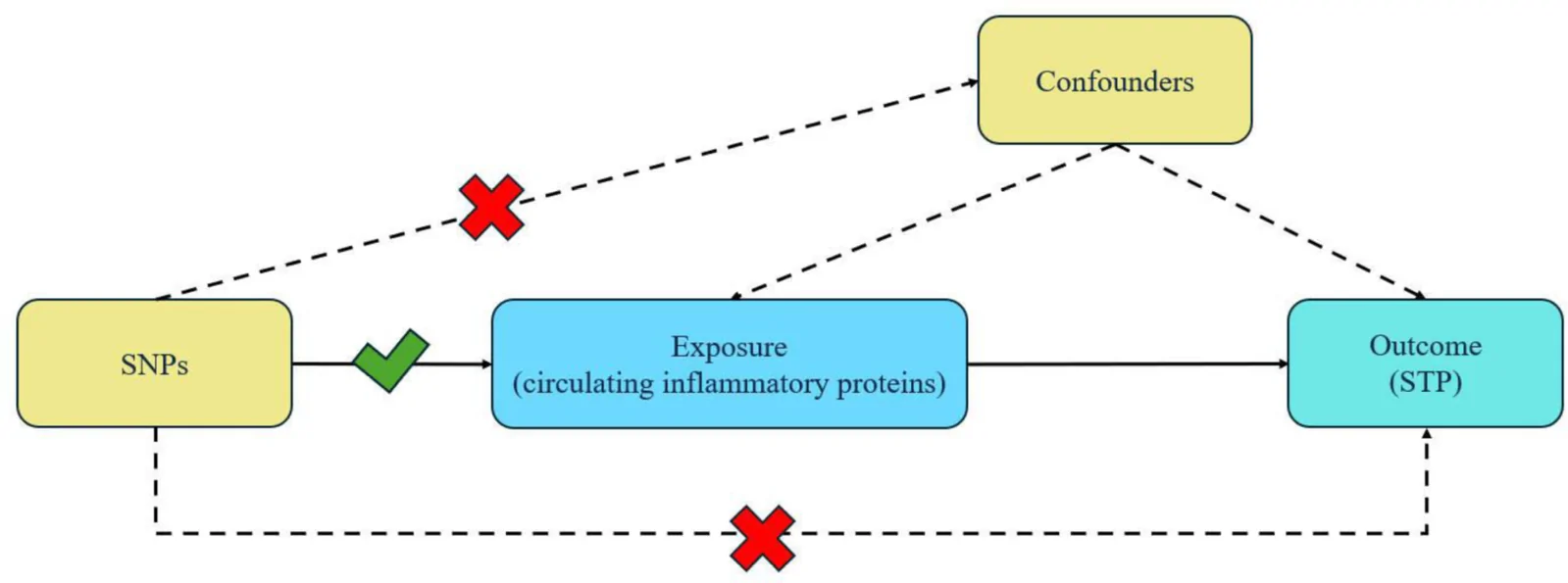
Study design overview and MR design hypothesis. SNPs = single nucleotide polymorphisms, STP = superficial thrombophlebitis, MR = Mendelian randomization.

Given that this study utilizes previously collected and publicly available GWAS data, additional ethical committee approval is not required.

### 2.2. Data sources and selection instrumental variables

For this study, data on circulating inflammatory proteins were sourced from the European Bioinformatics Institute, a global resource repository that extensively archives information related to GWAS. Researchers utilized the Olink Target platform to measure the levels of 91 plasma proteins in 14,824 individuals of European ancestry and conducted genome-wide protein quantitative trait loci (pQTL) analyses to determine the relationships between genetic variations of these proteins and their plasma levels, identifying a total of 180 pQTL (including 59 cis-pQTL and 121 trans-pQTL). The data for these 91 plasma inflammatory proteins, including the pQTL discoveries, are available in the European Bioinformatics Institute GWAS Catalog (accession numbers GCST90274758 to GCST90274848).^[13]^

The data for superficial phlebitis originated from the R11 release of the FinnGen Consortium (finngen_R11_I9_PHLETHROM). The FinnGen Consortium is a comprehensive national research initiative aimed at integrating genetic information from Finnish biobanks with digital health records from national registries to conduct extensive GWAS.^[14]^ Compared to the previous R10 release (412,181 participants), this version of GWAS encompassed 453,733 participants, analyzing 21,311,942 variants. Based on the International Classification of Diseases (ICD)-8 (451), ICD-9 (4519X|4518X), and ICD-10 (I80), and after excluding cases with specific ICD codes, a final cohort of 8602 cases and 392,860 controls was established (https://storage.googleapis.com/finngen-public-data-r11/summary_stats/finngen_R11_I9_PHLETHROM.gz). All GWAS summary statistics used in this study are from publicly available open-access databases, and the detailed data acquisition methods and access links are provided in the Data and Code Availability section of this manuscript.

In MR analysis, the rational selection of IVs is crucial for ensuring the validity and accuracy of causal inferences between circulating inflammatory proteins and STP. To achieve this, we adopted the following steps for screening:

Utilizing summary statistics from GWAS, we screened for significant single nucleotide polymorphisms (SNPs) among genetic variants associated with circulating inflammatory proteins. Considering the inherent characteristic of low heritability and extreme scarcity of strong-effect genome-wide significant loci (*P* < 5 × 10^−8^) for pQTL of inflammatory proteins, we set the initial significance level at *P* < 1 × 10^−5^, which is a widely accepted standard in pQTL-based MR studies, used to obtain sufficient IVs and ensure a strong correlation between the selected SNPs and circulating inflammatory proteins.^[8,12]^ To ensure the independence of the selected SNPs, we further applied exclusion criteria based on linkage disequilibrium (LD). By setting thresholds of *r^2^* = 0.001 and a physical distance threshold of kb = 10000, we excluded SNPs that were too close in physical distance and highly correlated statistically, thereby avoiding potential interference from LD in subsequent analyses.^[15]^ We used the “LDtrait”^[16]^ database to exclude SNPs associated with STP-related confounding factors (including varicose veins, thromboembolism, and vasculitis), with all excluded confounding loci and corresponding SNPs fully listed for each protein exposure (Supplementary Table 1, Supplemental Digital Content 1).^[5,6,17]^ If a factor was identified as a confounder, the corresponding SNP was removed. and We used the *F*-statistic to assess the strength of each SNP as an IV. The formula for calculating the *F*-statistic is *F* = *R*^2^(n–k–1)/k(1–*R^2^*), where n is the sample size of the exposed GWAS, *k* is the number of IVs included, and *R*^2^ is the proportion of variance explained by the SNP in the exposure database. The *F*-statistic is an important indicator of the association strength between IVs and the exposure factor. IVs with an *F*-value <10 were considered weak instrumental variables and were excluded from the MR analysis, with detailed information on all eligible SNPs including allele data, effect estimates, effect allele frequencies, and *F*-statistics fully documented for each circulating inflammatory protein (Supplementary Table 2, Supplemental Digital Content 2).

### 2.3. Statistical analysis

In the MR analysis, we employed the inverse-variance weighted (IVW) method as the primary research approach to explore the causal relationship between circulating inflammatory proteins and STP. When each genetic variant satisfies the assumptions of IVs, the IVW method can provide a consistent estimate of the causal effect of the exposure on the outcome.^[18]^ However, a single analytical method may face some limitations. Therefore, we also utilized MR-Egger regression, the Weighted Median method, the Simple Median method, and the Weighted Mode to supplement and validate the results.^[19]^ Additionally, we used the MR-PRESSO tool to test for the presence of horizontal pleiotropy, which can effectively identify and correct biases arising from confounding factors, thereby enhancing the accuracy of causal inference. We also applied the reverse MR method to assess whether the outcome could influence the exposure.

To ensure the robustness and reliability of the results and to detect potential sources of bias, we employed a series of sensitivity analysis methods, including the MR-Egger regression intercept test, Cochran’s *Q* test, and the Leave-One-Out (LOO) test. The MR-Egger regression intercept test was used to reexamine the presence of horizontal pleiotropy, with an intercept close to zero and a *P*-value >.05 indicating a weaker likelihood of horizontal pleiotropy.^[20]^ Cochran’s *Q* test was used to assess whether there was heterogeneity among the IVs, with a *P*-value >.05 indicating the use of the fixed-effects inverse-variance weighted method, and a *P*-value less than or equal to .05 indicating the use of the random-effects inverse-variance weighted method.^[21]^ The LOO method involves excluding each SNP as an instrumental variable1by one and then reestimati the causal effect based on the remaining SNPs, in order to assess the degree of influence of different SNPs on the causal relationship.

All analyses were conducted in *R* software (version 4.4.0) using the “TwoSampleMR” (version 0.6.4) and “MRPRESSO” (version 1.0) packages. To control the inflation of type I error caused by multiple comparisons for 91 circulating inflammatory proteins, we supplemented 2 mainstream multiple testing correction methods: Bonferroni correction, with the adjusted significance threshold set at *P* < .05/91 ≈ 5.49 × × 10^−4^ ; Benjamini-Hochberg (B-H) false discovery rate (FDR) correction, with the adjusted significance threshold set at FDR < 0.05.^[8]^ The full reproducible code for all analyses, including the multiple testing correction workflow, is available in the GitHub repository specified in the Data Availability Statement.

## 3. Results

In conducting MR analysis, we treated circulating inflammatory proteins as the exposure factor and used the IVW method to delve into the potential associations between 91 circulating inflammatory proteins and STP (Fig.2) After a rigorous screening process, we ultimately identified 149 SNPs associated with 5 key circulating inflammatory proteins, which were used as IVs for STP. Specifically, among these SNPs, 33 were related to neurotrophin–3 (NT–3), 23 were associated with osteoprotegerin (OPG), 31 were linked to vascular endothelial growth factor A (VEGF–A), 33 were correlated with IL–12B, and 29 were related to interleukin–8 (IL–8). The full statistical results of MR analysis for all 91 circulating inflammatory proteins, including Bonferroni-corrected and B-H FDR-adjusted *P* values, are documented, with full reproducible analysis code available in the specified GitHub repository (Supplementary Table 3, Supplemental Digital Content 3).

**Figure 2.**
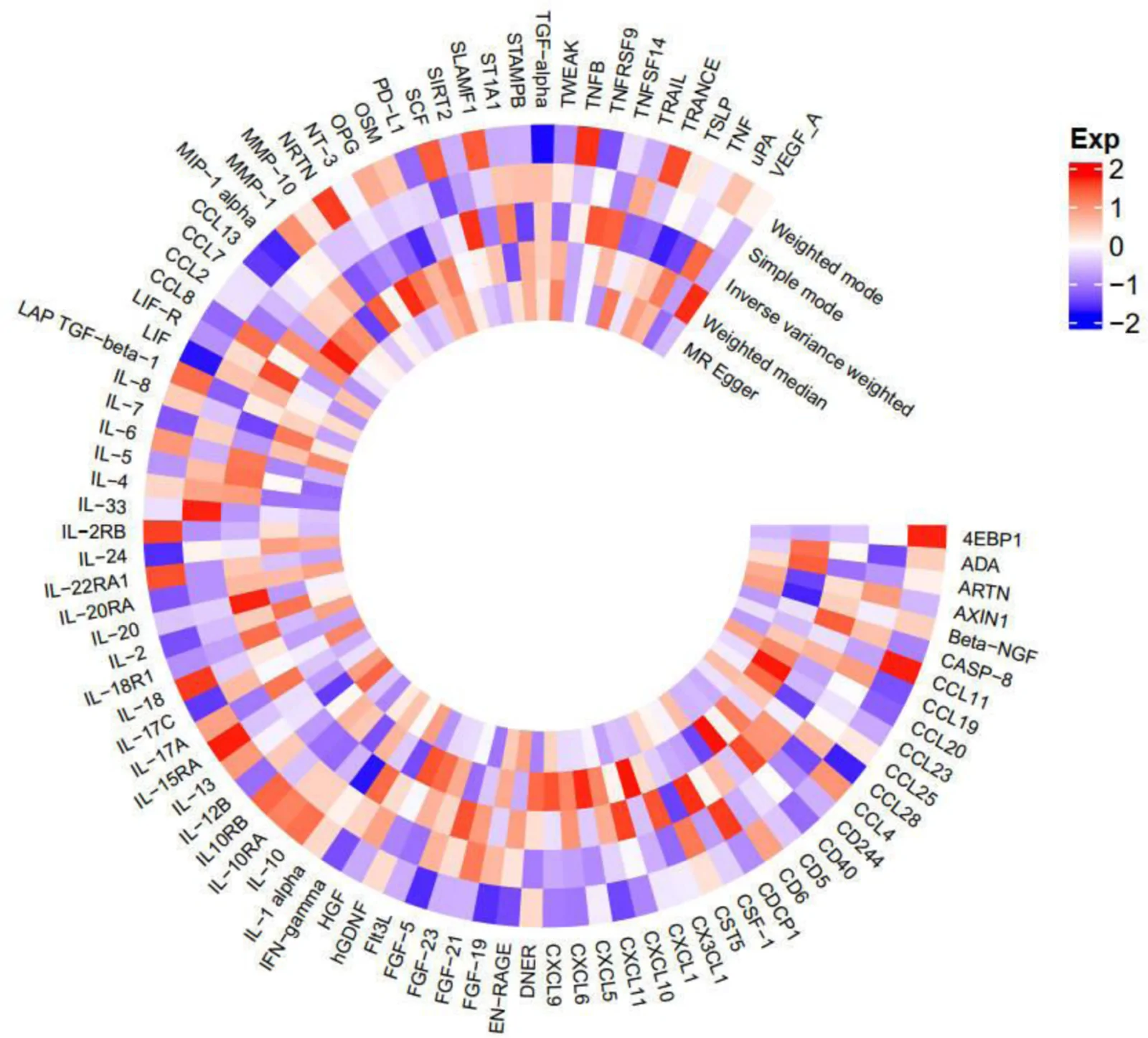
Comparison of the *p*-values for circulating inflammatory proteins related to STP. STP = superficial thrombophlebitis.

According to the IVW results of the MR analysis (Fig. 3), we observed nominal associations between 5 circulating inflammatory proteins and STP: neurotrophin-3 (NT-3) (OR = 1.132, 95%CI: 1.024–1.252, *P* = .016), OPG (OR = 1.120, 95%CI: 1.013–1.238, *P* = .027), and vascular endothelial growth thrombophlebitis factor A (VEGF-A) (OR = 1.068, 95%CI: 1.008–1.132, P = .027) were positively associated with STP risk, while IL-12B (OR = 0.917, 95%CI: 0.858–0.980, *P* = .011) and IL-8 (OR = 0.905, 95%CI: 0.824–0.993, *P* = .035) were negatively associated with STP risk (all raw *P* < .05), with full causal effect estimates including beta values, standard errors, odds ratios, and 95% confidence intervals across all complementary MR methods (MR-Egger regression, weighted median, simple mode, weighted mode) provided for each protein (Supplementary Table 4, Supplemental Digital Content 4). However, after multiple testing correction, none of the above associations reached the preset significance threshold: all FDR values were > 0.05 after B-H FDR correction, and all *P* values were higher than 5.49 × 10^−4^ after Bonferroni correction. It should be noted that the direction of the causal effects of the 5 proteins was completely consistent across MR-Egger regression, weighted median, weighted mode, and other complementary analysis methods, with no significant heterogeneity or horizontal pleiotropy observed in sensitivity analyses, indicating a stable trend of association.

**Figure 3.**
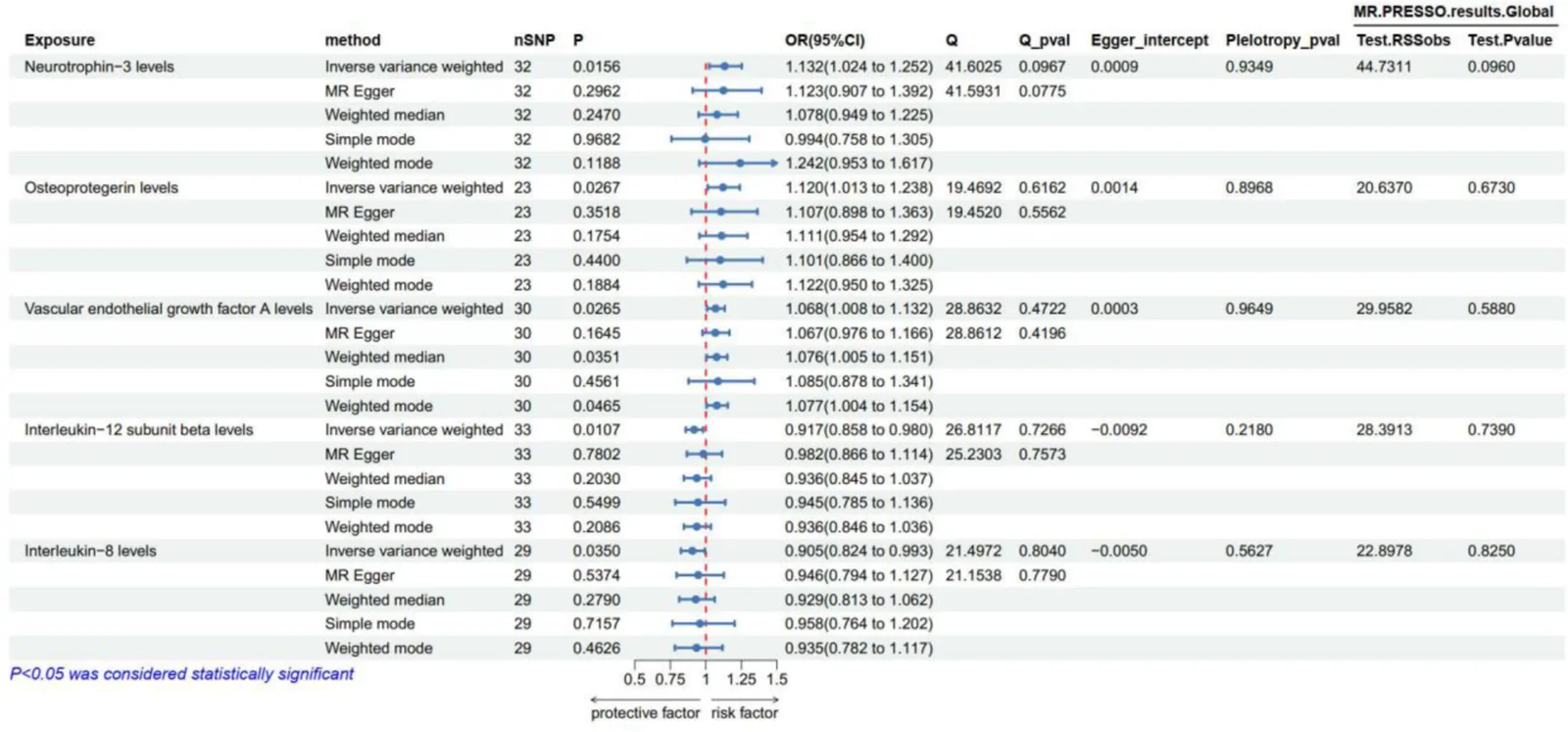
Associations between circulating inflammatory proteins and STP risk. STP = superficial thrombophlebitis. NT-3 = neurotrophin-3, VEGF-A = vascular endothelial growth thrombophlebitis factor A.

A series of sensitivity analyses were conducted to verify the robustness of the causal estimates (Fig. 3). Cochran’s *Q* test showed no significant heterogeneity among instrumental variables (*P* > .05), thus the fixed-effects inverse variance weighted method was used for the primary analysis. The MR-Egger regression intercept test confirmed no significant horizontal pleiotropy (intercept close to zero, all *P* > .05), consistent with the results of the MR-PRESSO test. In the MR-PRESSO test, no outliers were detected for any SNPs (*P* > .05), further confirming the absence of horizontal pleiotropy. Additionally, the funnel plot (Supplementary Figure 1, Supplemental Digital Content 5) did not show any significant asymmetry, further suggesting that horizontal pleiotropy can be considered a negligible factor. Meanwhile, no potential outliers were observed in the scatter plots (Supplementary Figure 2, Supplemental Digital Content 6). The results of the LOO analysis showed that the removal of any individual SNP did not significantly affect the overall results (Supplementary Figure 3, Supplemental Digital Content 7), robustly validating the stability of the associations revealed by this MR analysis. To further verify the robustness of the core results, we rescreen IVs using the strict genome-wide significant threshold of *P* < 5 × 10^−8^ and performed a supplementary sensitivity analysis. The results showed that although the statistical power of some proteins decreased and the *P* values increased due to the reduced number of IVs, the direction of causal effects between the 5 core inflammatory proteins and STP was completely consistent with the original core analysis, with no flip of effect direction, further confirming that the core association trend of this study is robust and reliable and is not caused by weak instrument bias introduced by the lenient initial threshold. Furthermore, reverse MR analysis showed no significant causal effect of genetically predicted STP on the circulating levels of 5 core inflammatory proteins (all raw *P* > .05), which ruled out the potential reverse causality between exposure and outcome in this study. Full multiple testing corrected results for all 91 proteins are provided in Supplementary Table 3, Supplemental Digital Content 3.

## 4. Discussion

Utilizing the MR research methodology, our study, based on an extensive review of the literature, represents the first in-depth exploration of the potential associations between 91 circulating inflammatory proteins and STP. We fully leveraged publicly available data on circulating inflammatory proteins from GWAS databases and integrated it with the latest data on STP from the R11 release of the FinnGen consortium. In our in-depth analysis, we identified nominal suggestive associations between NT-3, OPG, and VEGF-A and increased STP risk. Conversely, IL-8 and IL-12B showed nominal inverse associations with STP. As a hypothesis-generating MR study, the core objective of this research is to screen potential inflammatory protein targets associated with STP, and the identified associations need to be further verified by subsequent clinical and functional studies. After strict multiple testing correction, the above associations did not reach the preset statistical significance threshold, indicating that the statistical robustness of the potential associations still needs to be confirmed by large-sample studies in the future. However, the consistent effect direction across multiple analytical methods suggests that these proteins still have potential biological rationality for in-depth exploration and provide a clear research direction for subsequent verification studies on the inflammatory mechanism of STP.

As a member of the neurotrophin family, NT-3 exerts its functions through interactions with tyrosine kinase receptors on the cell membrane and the common neurotrophin receptor p75NTR.^[22]^ Depending on the receptor it binds to, NT-3 elicits diverse effects, not only promoting neuronal growth and differentiation but also playing a pivotal role in inflammatory responses. Macrophages play a central role in the inflammatory process and secrete NT-3 during inflammatory reactions.^[23]^ Studies have shown that NT-3 promotes the production of extracellular matrix proteins, thereby enhancing the activity of fibroblasts.^[24]^ This reveals the significant role of NT-3 in the fibrotic process induced by inflammatory responses. Furthermore, extracellular matrix proteins contribute to thrombus formation by promoting platelet aggregation and providing adhesion sites.^[25]^ The interplay between inflammation and thrombosis collectively exacerbates the progression of STP, which aligns with our nominal observation of a positive association between NT-3 and STP risk.

OPG is a secreted glycoprotein that plays a central role in bone turnover and has traditionally been associated with various bone-related diseases.^[26]^ Increasingly, studies have found that the role of OPG extends beyond bone disorders, with close links to oncology and cardiovascular diseases. Epidemiological research suggests that serum OPG levels can serve as a biomarker for predicting cardiovascular complications.^[27]^ During inflammatory responses, OPG activates NF-κB, inducing the expression of adhesion molecules, which in turn facilitates the adhesion and infiltration of leukocytes onto the vascular wall, ultimately leading to endothelial dysfunction.^[28]^ While studies have reported that elevated OPG levels may indicate acute inflammatory responses in cardiovascular diseases such as coronary heart disease, it is noteworthy that there is currently a lack of biological and epidemiological evidence linking OPG to STP. Further research is needed to delve into the specific mechanisms underlying the association between OPG levels and STP.

VEGF-A is a pivotal member of the vascular endothelial growth factor (VEGF) family, exerting its crucial role through binding to tyrosine kinase cellular receptors. The VEGF receptor family primarily consists of 3 types: VEGFR-1, VEGFR-2, and VEGFR-3.^[29]^ VEGFR-1 and VEGFR-2 are predominantly expressed on the surface of vascular endothelial cells, whereas VEGFR-3 is mainly expressed on endothelial lymphocytes.^[30,31]^ When VEGF-A binds to VEGFR-1, it activates the phosphoinositide 3-kinase/protein kinase B (PI3K/Akt) signaling pathway, thereby promoting the migration of inflammatory cells, such as monocytes, macrophages, and polymorphonuclear leukocytes. Additionally, the activation of VEGFR-1 stimulates the release of various pro-inflammatory cytokines by inflammatory cells, further exacerbating the inflammatory response.^[32]^ A MR study has shown that VEGF-A levels are positively correlated with the risk of varicose veins, which is a significant pathogenic factor for STP.^[8]^ Therefore, we hypothesize that VEGF-A may promote the occurrence and development of STP by influencing the pathogenesis of varicose veins, providing a new perspective and approach for the prevention of STP.

IL-8 is a neutrophil-activating cytokine produced by macrophages and various tissue cells in response to inflammatory stimuli.^[33]^ It binds to G-protein-coupled receptors CXCR1 and CXCR2, directing neutrophils to migrate towards sites of injury and inflammation.^[34]^ Although IL-8 is recognized as a pro-inflammatory cytokine, our study observed a nominal inverse association between IL-8 and STP. An animal experiment revealed that IL-8 promotes thrombus formation in the initial phase (within 4 days) but subsequently (after 8 days) accelerates thrombolysis and promotes neovascularization.^[35]^ A previous study involving 68 clinical patients with STP showed that levels of inflammatory markers were negatively correlated with the recanalization rate of superficial venous thrombosis. During a 12-month follow-up, the levels of most inflammatory markers declined significantly, but IL-8 levels did not change markedly compared to the acute phase.^[36]^ We speculate that IL-8 has multiple roles in STP, with primary involvement in thrombolysis and neovascularization processes during the chronic phase. Its level does not decrease continuously like other inflammatory markers, and further clinical or experimental data are needed to substantiate this observation.

In our MR analysis, IL-12B showed a nominal inverse association with STP risk. However, it is noteworthy that IL-12B constitutes the p35 and p40 subunits of IL-12, which promotes Th1 cellular immune responses.^[37]^ As the primary effector molecule of Th1 cells, interferon-*γ* enhances the phagocytic and bactericidal capabilities of macrophages and indirectly exacerbates inflammatory responses in the vascular wall by activating other immune cells, such as natural killer cells and cytotoxic *T* lymphocytes.^[38]^ Although there are currently limited studies on the relationship between IL-12 and the risk of STP, multiple studies have demonstrated the inflammatory damage effects of IL-12 on different tissues, which seemingly contradicts our findings. Additionally, MR studies by Weronica et al and Meng et al also observed phenomena where the research results of IL-12B contradict actual clinical situations.^[39,40]^ We speculate that this may be due to the impact of genetic variations on outcomes being modulated by various factors such as environmental factors, population characteristics, and therapeutic interventions. In MR studies, such complex interactions may be difficult to accurately identify and control, leading to discrepancies between research findings and clinical realities. Therefore, despite our research results indicating that IL-12B may play a role in the pathogenesis of STP, the reliability of these results may be compromised by numerous unverified hypotheses.

In this study, we utilized a 2-sample MR approach for the first time to delve into the potential associations between circulating inflammatory proteins and STP. Compared to traditional observational analysis methods, the MR approach is more effective in mitigating the influence of confounding factors and reverse causality. After conducting tests for pleiotropy using MR-PRESSO and MR-Egger regression intercept tests, our study identified nominal associations between IL-8, IL-12B, NT-3, OPG, and VEGF-A and STP, with consistent effect directions across complementary methods, though these associations did not survive multiple-testing correction.

However, this study also has certain limitations. Firstly, the GWAS summary data used in this study were all derived from European ancestry populations; thus, the applicability of the study results to other ethnic populations may be limited. Secondly, a significance threshold of *P* < 1 × 10^−5^ was set when screening instrumental variable SNPs for inflammatory proteins, which is a widely accepted standard in pQTL-based MR studies considering the low heritability and scarcity of strong-effect pQTLs for inflammatory proteins. We further strictly retained only SNPs with an *F* statistic > 10 (average *F* statistic = 28.7, range: 12.3–45.6) to completely eliminate weak instrument bias and verified the robustness of the core results through supplementary sensitivity analysis with a strict genome-wide significant threshold of *P* < 5 × 10^−8^, which confirmed the consistent effect direction of the core associations. Thirdly, this study is a hypothesis-generating MR study based on public GWAS summary data. After multiple testing correction, the potential associations between the 5 screened inflammatory proteins and STP did not reach the strict statistical significance threshold, and no in vitro/in vivo functional experiments or RT-qPCR validation in clinical samples were performed for these core proteins. The statistical robustness and biological functions of the identified targets still need to be further verified by large-sample clinical cohort studies and functional experiments in the future. Fourthly, the potential causal relationships between some circulating inflammatory proteins and STP are reported here for the first time, and the specific biological mechanisms of these proteins in the pathogenesis of STP cannot be fully elucidated by the MR approach alone, which warrants further in-depth exploration in subsequent studies.

## 5. Conclusion

In conclusion, this hypothesis-generating MR study explored the potential associations between 91 circulating inflammatory proteins and STP and identified 5 proteins (NT-3, OPG, VEGF-A, IL-12B, and IL-8) with nominal suggestive associations with STP risk, though none remained significant after correction for multiple testing. These findings provide candidate inflammatory proteins for future validation and offer directions for further investigations into the inflammatory mechanisms underlying STP.

## Acknowledgments

We would like to thank the participants and researchers of the FinnGen study and the IEU Open GWAS Project. We sincerely thank all the researchers who contributed valuable genetic linkage statistical data to this study.

## Author contributions

**Conceptualization:** Qingzhi Liu, Yannan Wang.

**Data curation:** Qingzhi Liu, Weiyue Chen, Xiangyu Wang, Hong Mao, Boxun Chen.

**Formal analysis:** Qingzhi Liu, Weiyue Chen.

**Funding acquisition:** Qingzhi Liu, Yannan Wang.

**Methodology:** Qingzhi Liu, Weiyue Chen, Yannan Wang.

**Validation:** Qingzhi Liu, Xiangyu Wang, Hong Mao, Yannan Wang.

**Visualization:** Weiyue Chen, Boxun Chen.

**Project administration:** Youwen Zhang, Jiaxiang Xu, Yannan Wang.

**Supervision:** Yannan Wang.

**Writing – original draft:** Qingzhi Liu, Weiyue Chen.

**Writing – review & editing:** Hong Mao, Boxun Chen, Yannan Wang.














