# Supplementary material for: Exploring causal relationships between circulating inflammatory proteins and superficial thrombophlebitis: A Mendelian randomization study
Source: Medicine (Baltimore). 2026 Sep 25;105(39):e50749. doi: 10.1097/MD.0000000000050749 (PMC13619184; doi:10.1097/MD.0000000000050749)
Supplement: Supplementary file 2 [file medi-105-e50749-s006.pdf]

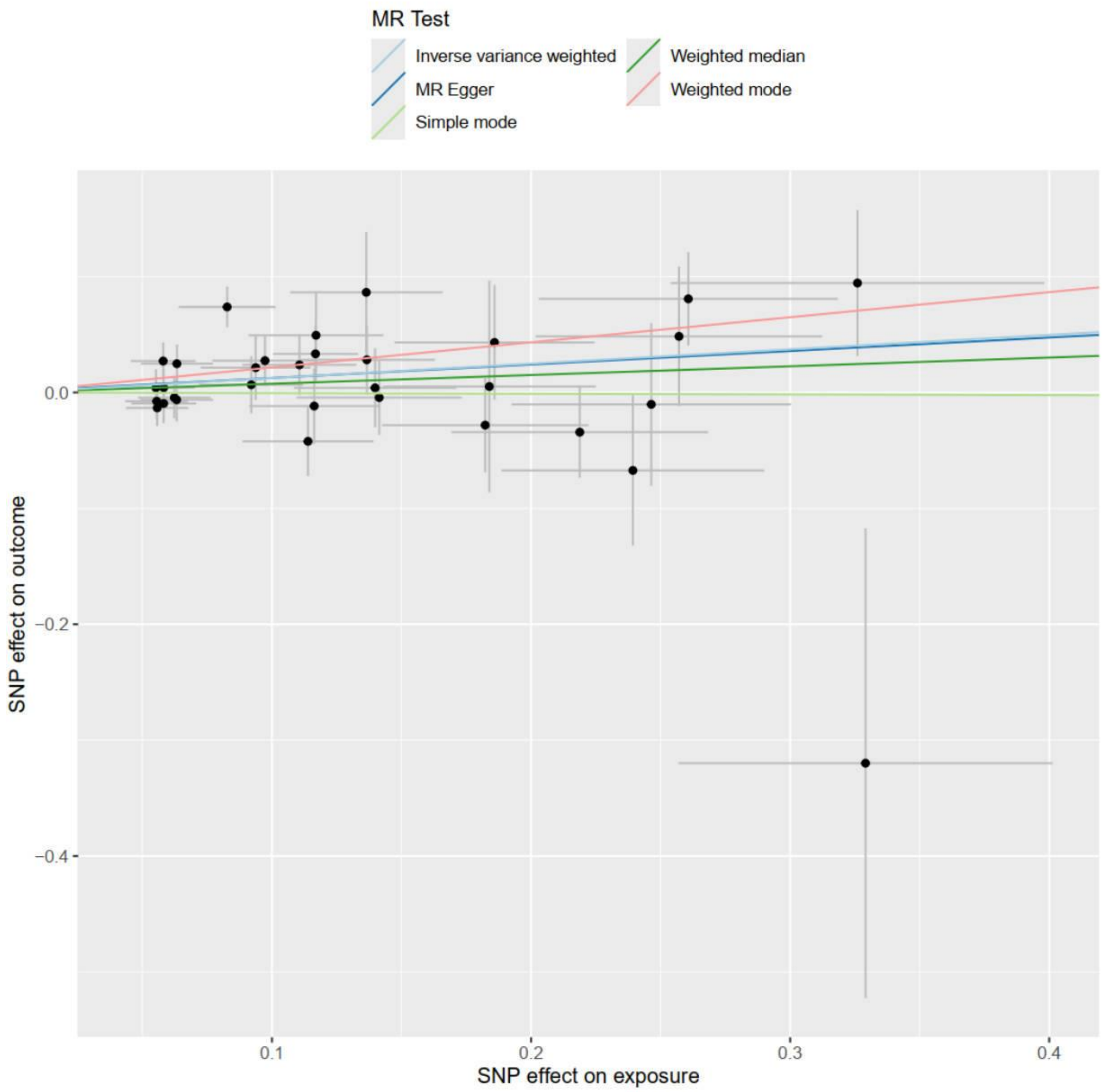

SNP effect on Neurotrophin-3 measurement

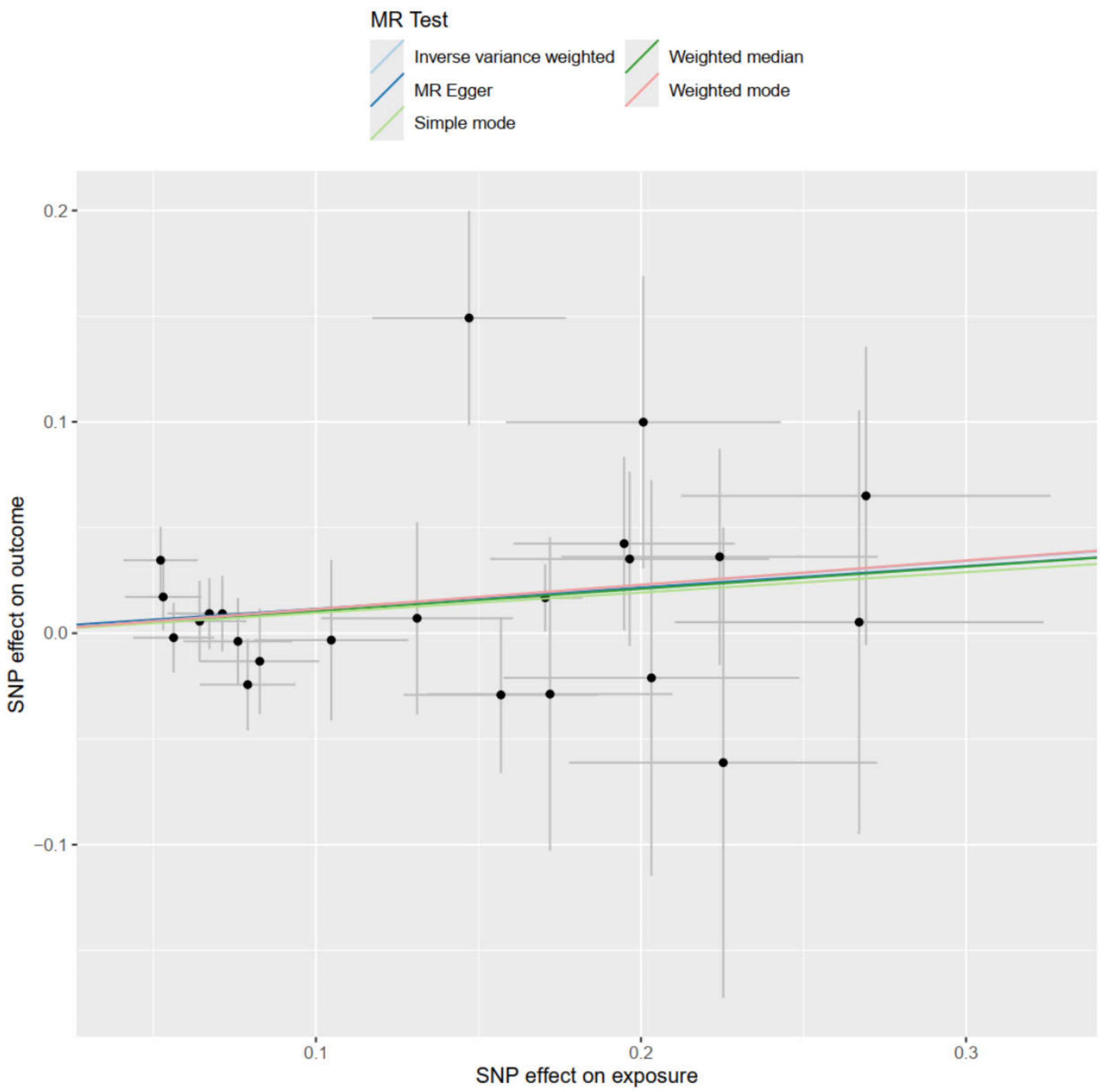

SNP effect on Osteoprotegerin measurement

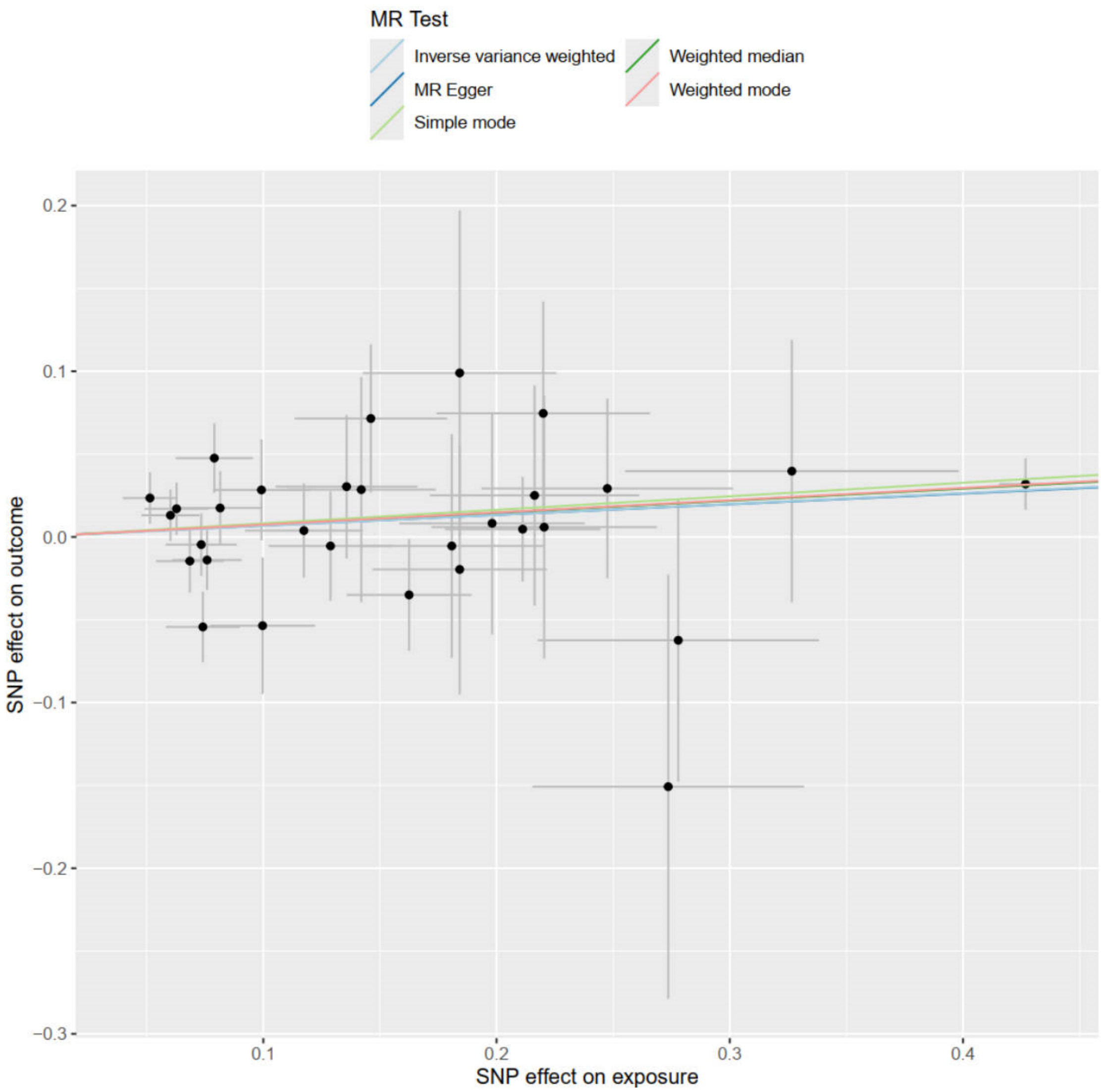

SNP effect on Vascular endothelial growth factor A measurement

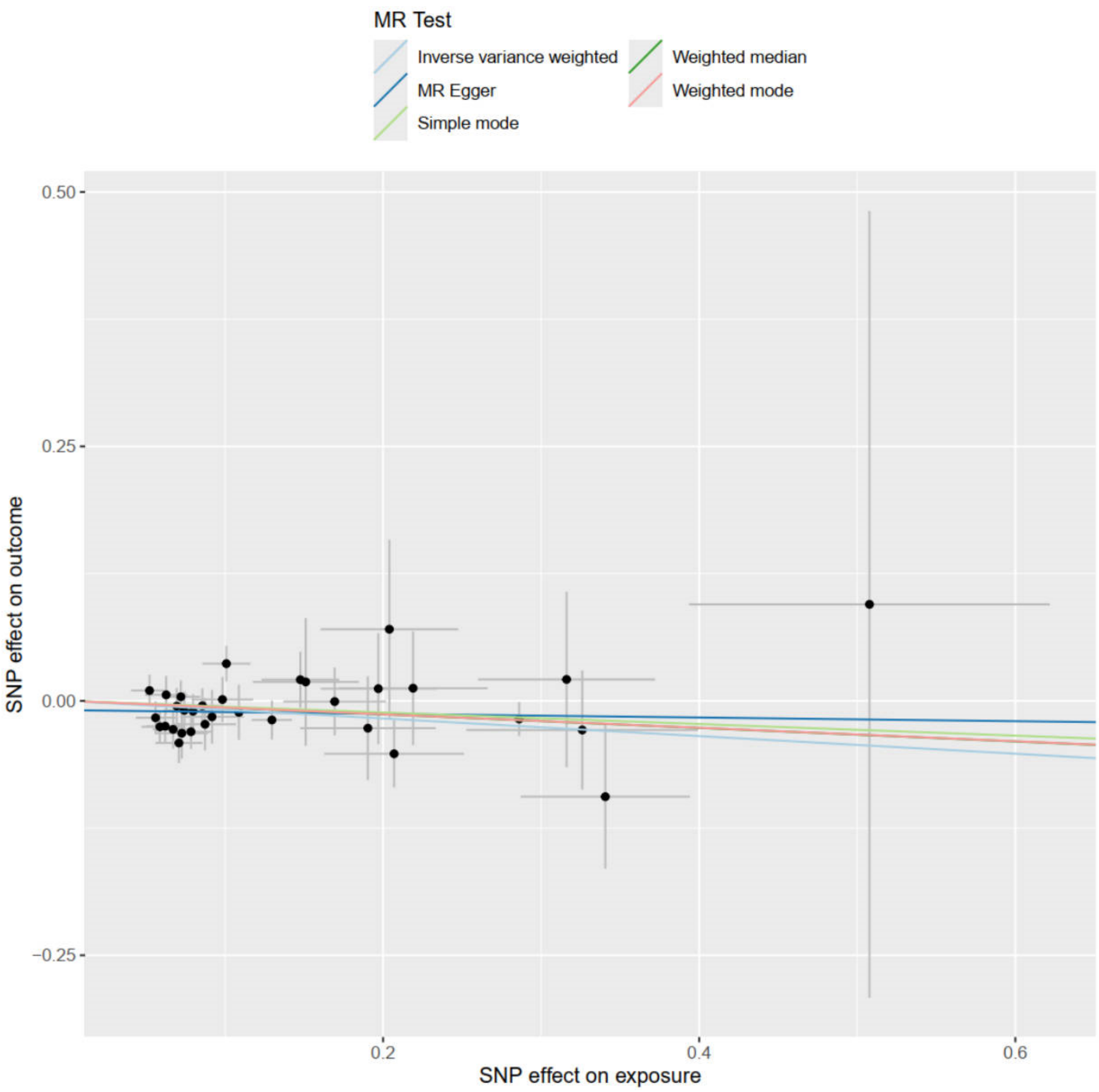

SNP effect on Interleukin-12 subunit beta measurement

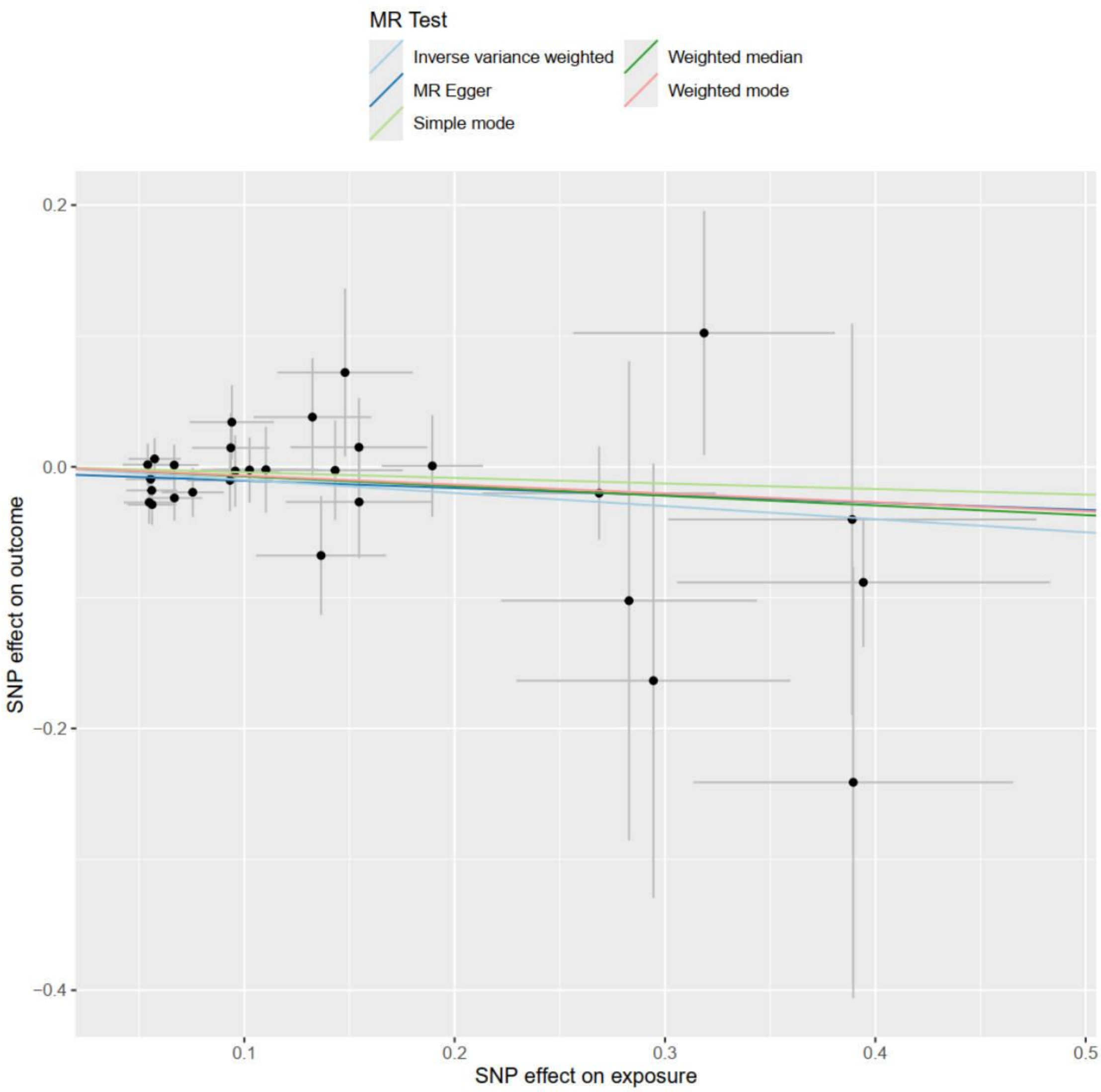

SNP effect on Interleukin-8 measurement
