# Supplementary material for: Exploring causal relationships between circulating inflammatory proteins and superficial thrombophlebitis: A Mendelian randomization study
Source: Medicine (Baltimore). 2026 Sep 25;105(39):e50749. doi: 10.1097/MD.0000000000050749 (PMC13619184; doi:10.1097/MD.0000000000050749)
Supplement: Supplementary file 3 [file medi-105-e50749-s007.pdf]

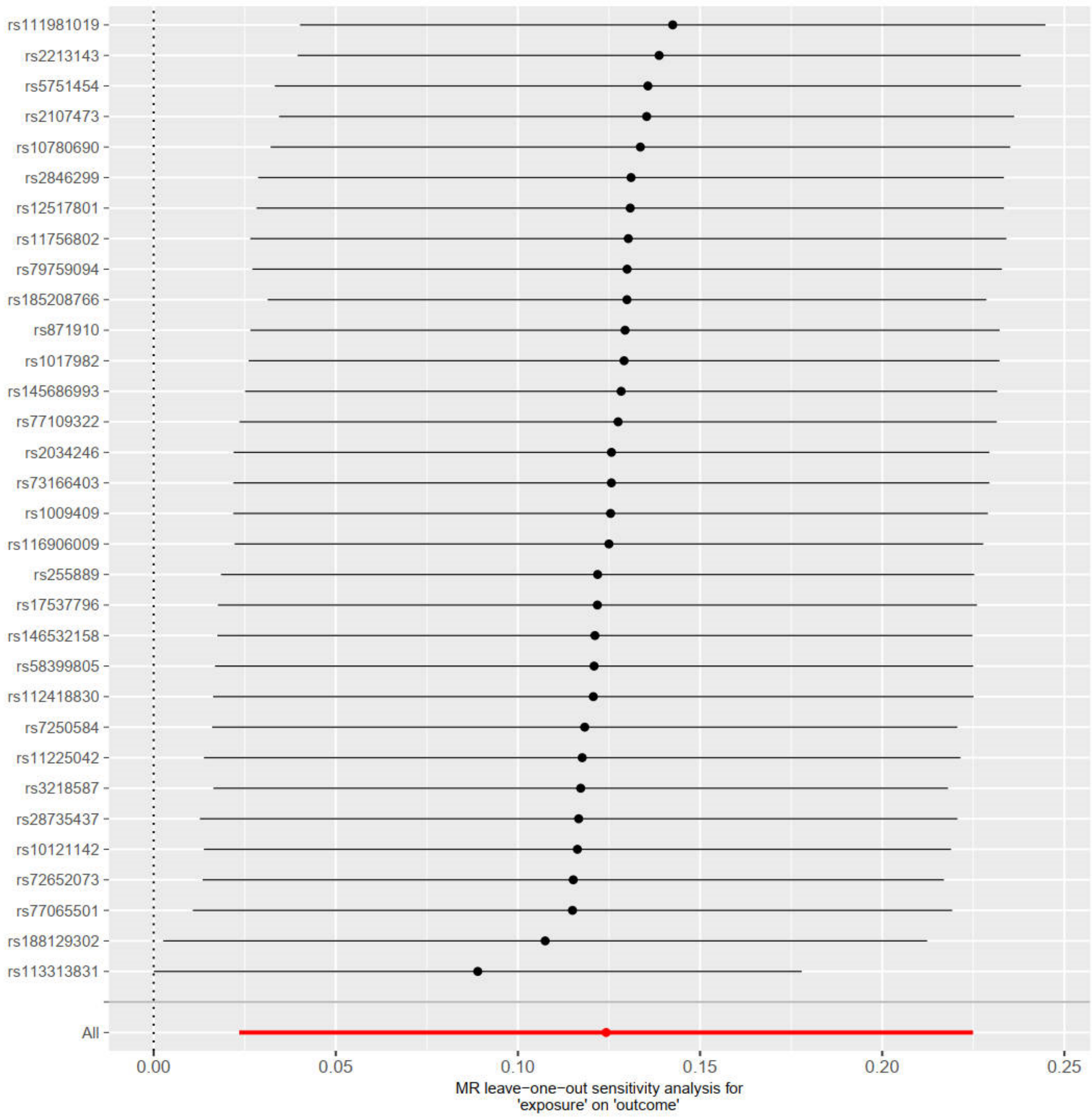

MR leave-one-out sensitivity analysis for
'Neurotrophin-3 measurement' on 'outcome'

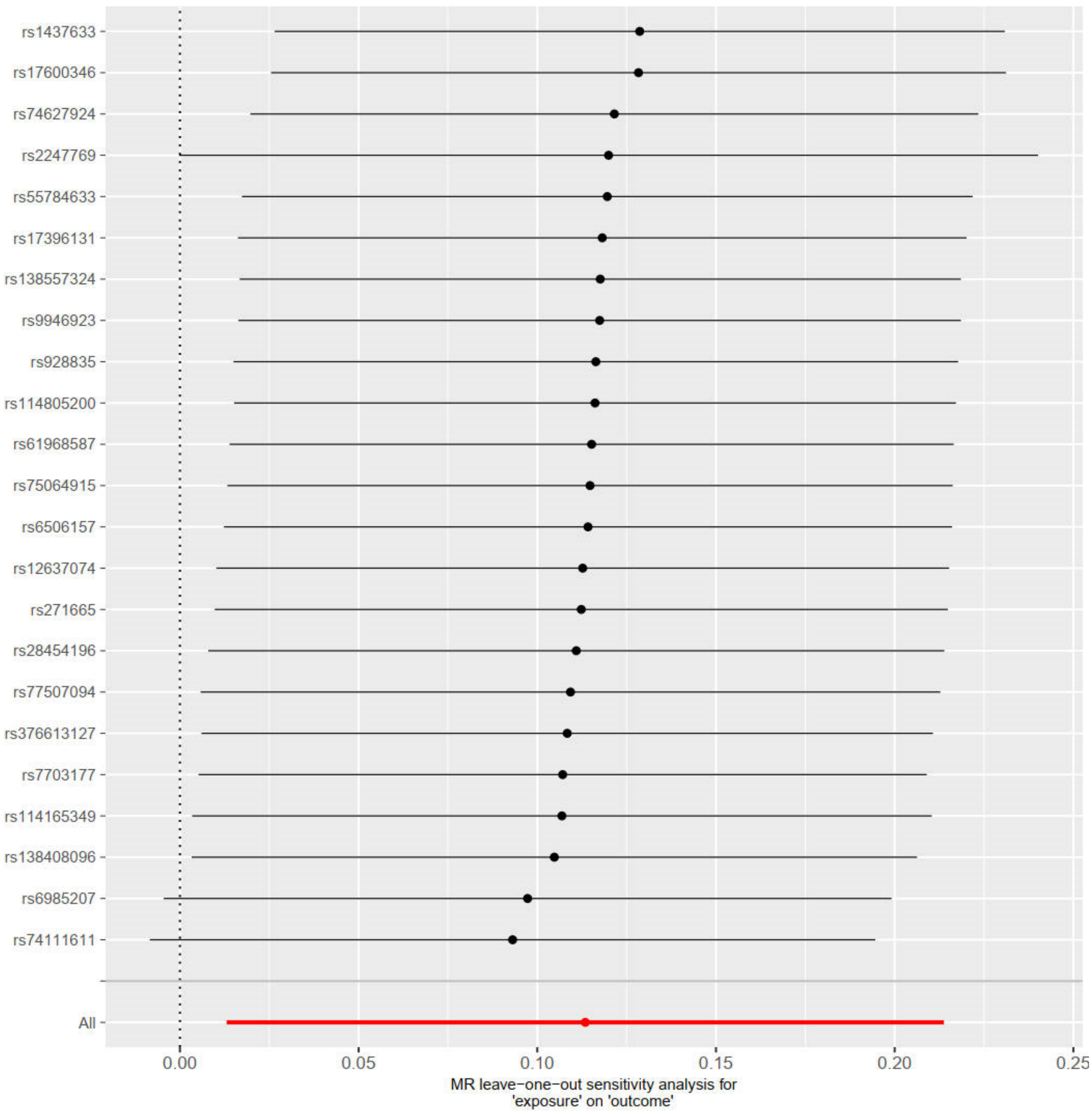

MR leave-one-out sensitivity analysis for
'Osteoprotegerin measurement' on 'outcome'

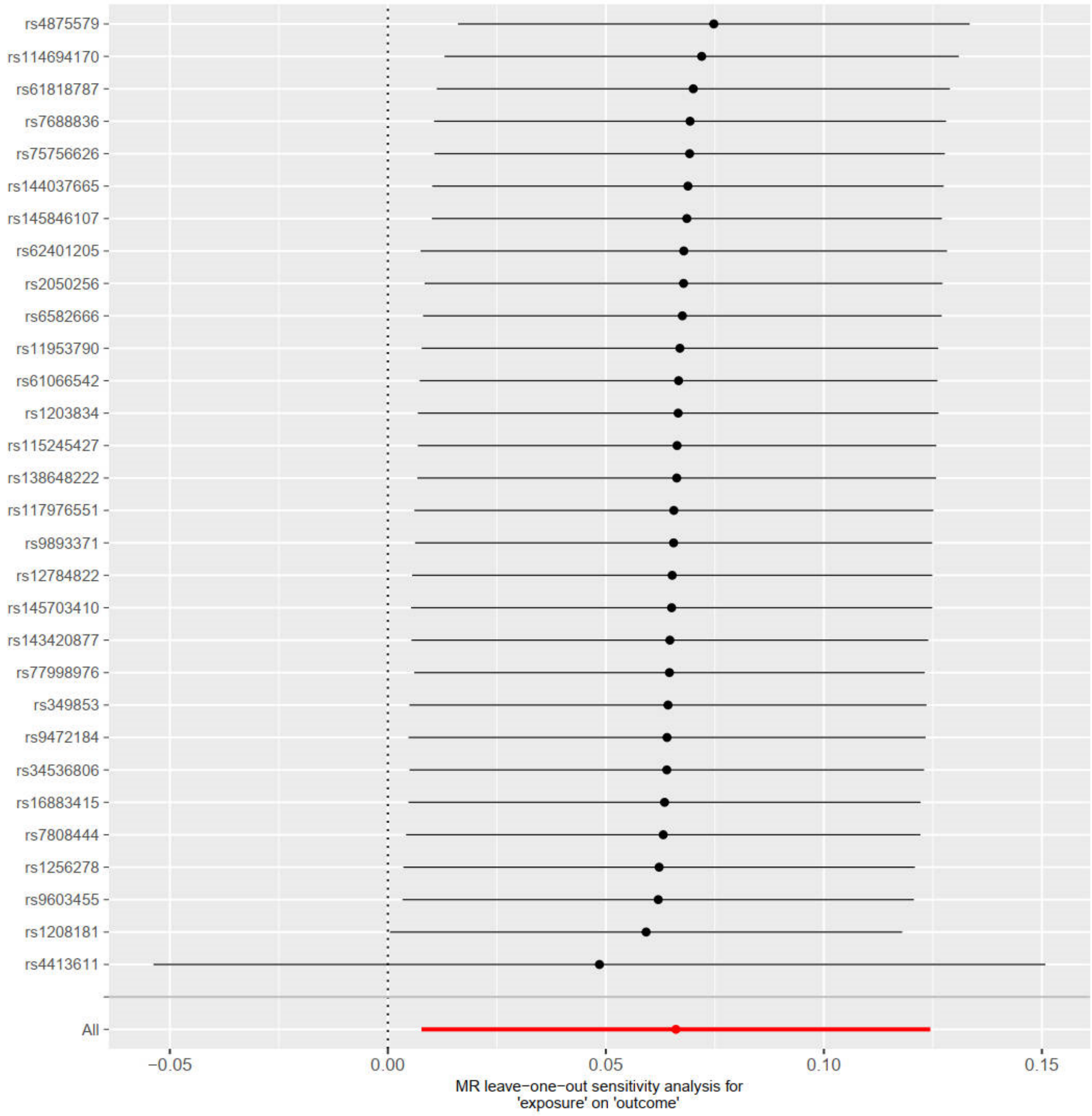

MR leave-one-out sensitivity analysis for
'Vascular endothelial growth factor A measurement' on 'outcome'

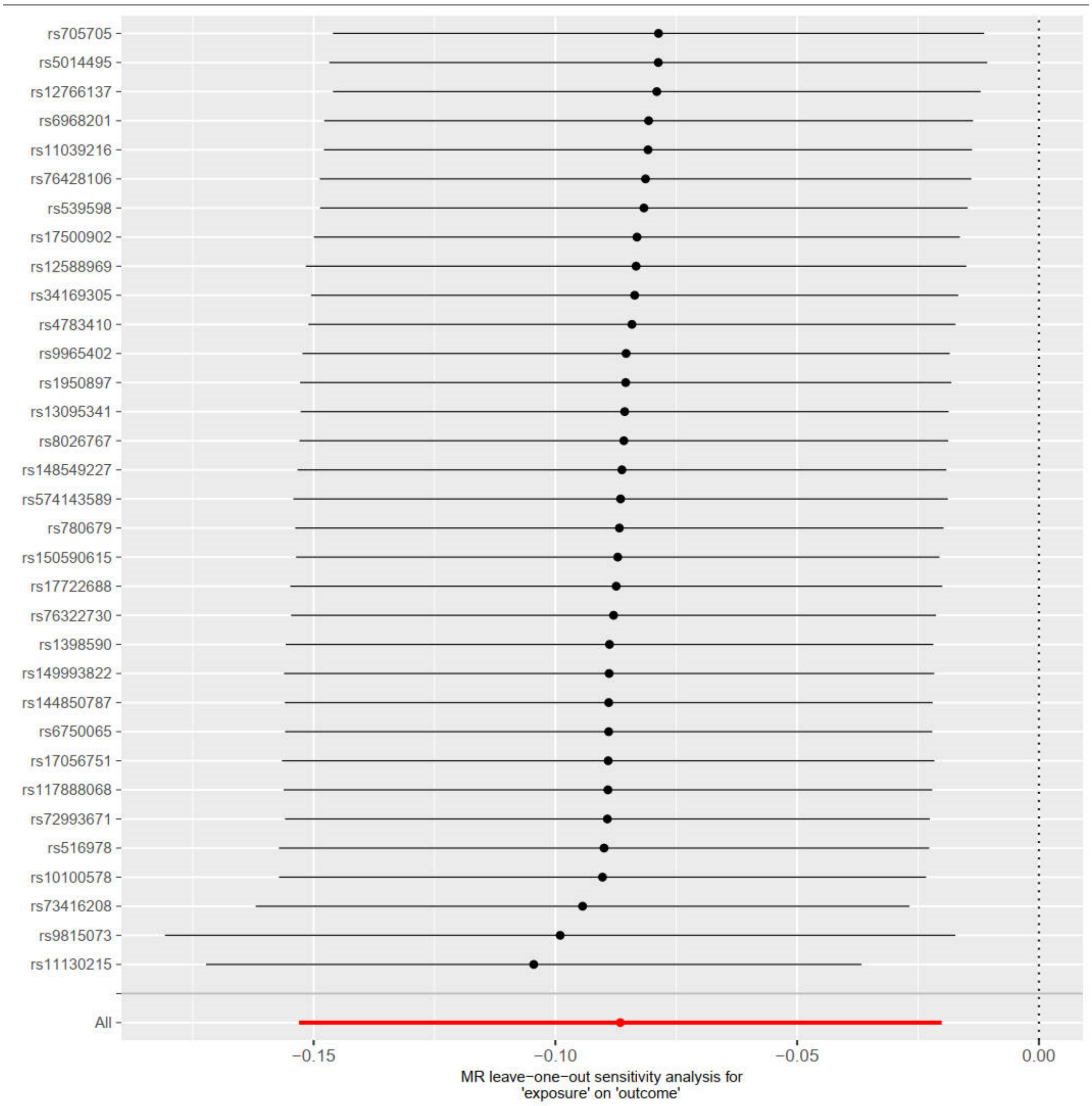

MR leave-one-out sensitivity analysis for
'Interleukin-12 subunit beta measurement' on 'outcome'

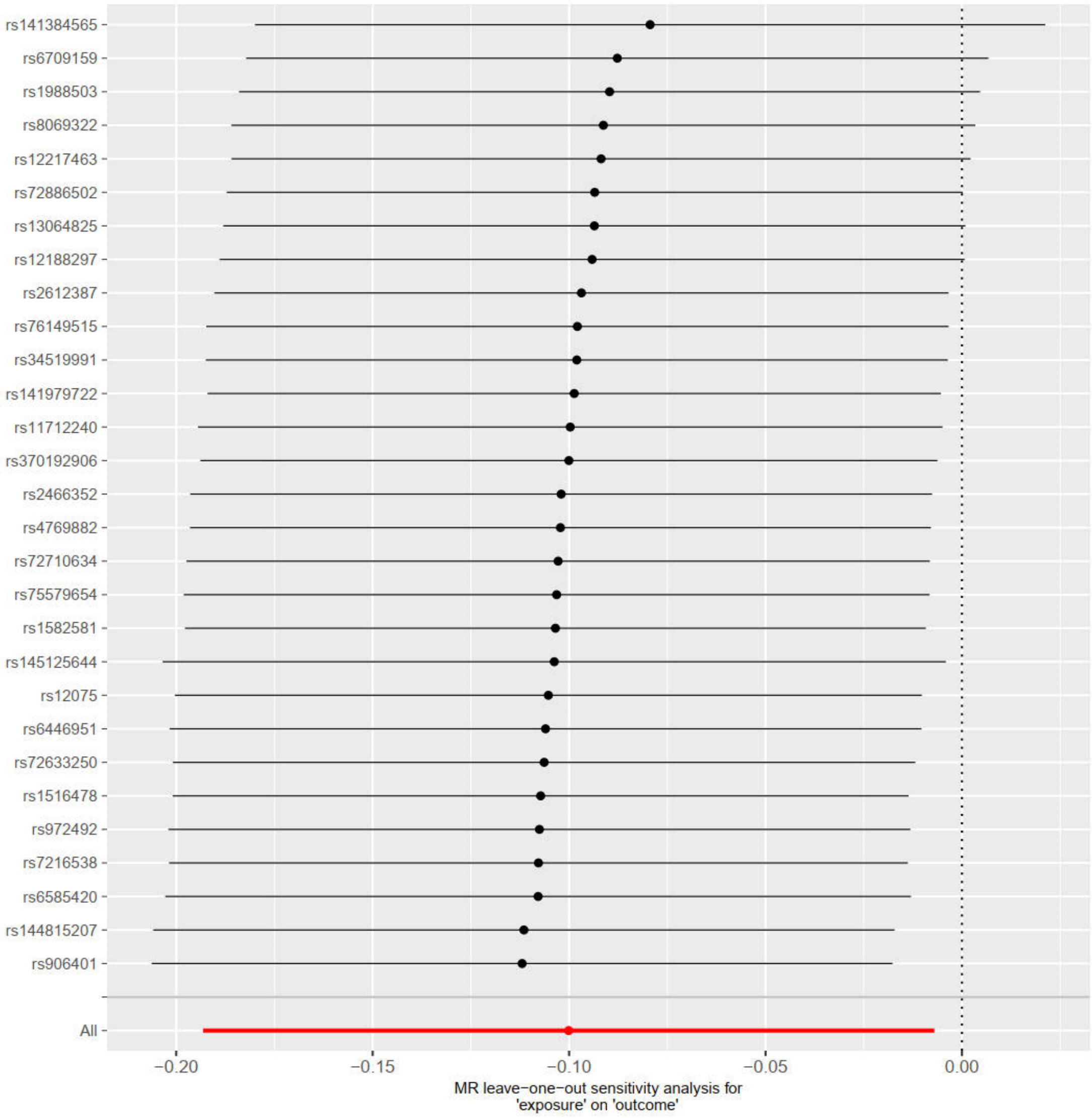

MR leave-one-out sensitivity analysis for
'Interleukin-8 measurement' on 'outcome'
